# Discovering Pathologies in the Anatomy Lab: The Case of Brachial Plexopathy Mimicking Neurological Thoracic Outlet Syndrome

**DOI:** 10.51894/001c.14179

**Published:** 2020-10-30

**Authors:** Ryley Mancine, Paul Kowalski, William McMillan, Nicole Geske, Loro Kujjo

**Affiliations:** 1 Department of Anatomy, Division of Human Anatomy Michigan State University College of Osteopathic Medicine; 2 Department of Physiology Michigan State University; 3 Department of Anatomy, Division of Human Anatomy Michigan State University

**Keywords:** suprascapular nerve, subclavicular lipoma, lipoma, ntos, brachial plexopathy

## Abstract

**CONTEXT:**

Well-established human anatomy labs with access to expert faculty are exceedingly valuable tools to medical student education. In this manuscript, we detail an infero-lateral subclavicular lipoma which was discovered as a result of the utilization of both those labs and expert faculty. This lipoma may have caused brachial plexopathy or may serve as an unusual cause of neurologic thoracic outlet syndrome (NTOS) due to the location of the mass.

**EDUCATIONAL CASE PRESENTATION:**

During prosection of a donor in the human anatomy lab, a mass was discovered by a medical student. This medical student utilized the human anatomy lab faculty members and resources to identify the mass as a lipoma. The lipoma compressed the lateral cord of the brachial plexus and the suprascapular nerve, but no diagnosis of NTOS or brachial plexopathy was made during the life of the donor, nor was any surgical intervention indicated. Removal of the lipoma immediately relieved stress upon the nerves. Histochemical analysis confirmed the diagnosis of a lipoma and demonstrated almost only mature adipocytes.

**CONCLUSION:**

The authors concluded that the lipoma of this patient was not identifiable with computerized tomography imaging modalities, despite ultrasound demonstrating a hyperechoic outline of the mass in the cadaver of the patient. It is very likely that this lipoma had not been diagnosed previously due to the atypical location of the tumor. Equally, typical surgical methods associated with brachial plexopathy or NTOS treatment would be difficult or more complicated, due to the lateral and inferior location of the lipoma. Physicians treating thoracic outlet syndrome-type symptoms without resolution should consider potential non-malignant obstruction located outside the thoracic outlet, toward the extremity. Deep palpatory methods and physical therapy should be considered until diagnosis is certain, as ultrasound would be difficult and typical transaxillary surgical methods would be nonhelpful. Medical students and early-career residents and physicians should be aware of the resources provided to them via campus human anatomy laboratories which they may utilize to further their understanding and knowledge of specific pathologies.

## INTRODUCTION

At Michigan State University College of Osteopathic Medicine (MSUCOM), all students are currently required to take an observations-based human anatomy laboratory course at the start of their medical coursework. During subsequent semesters, preclerkship (usually first and second year) and clerkship (final two years, i.e., third and fourth year) medical students can also enroll in elective anatomy courses which allow hands-on prosection (i.e., observed dissection) of select regions or parts of preserved human cadavers. Cadavers are used for teaching in observations-based medical and undergraduate courses. The MSUCOM prosection and gross anatomy teaching labs all operate in compliance with Willed Body Programs policy guidelines.

In this article, the authors narratively describe their identification and post-mortem diagnosis of a tumorous mass (i.e., lipoma), during a routine prosection of a human cadaveric donor. The authors will highlight the academic value of in-person medical school prosection and gross anatomy laboratories learning.

Lipomas (i.e., slow-growing fatty lumps) in the thoracic outlet are very rare, with those reported as infraclavicular (i.e., located below the clavicle) in the literature even more scarce.^1^ During a 2017 case-series and literature review by Graf et al., only 12 brachial plexus tumors had been identified over 10 years, with only one diagnosed as infraclavicular.^1^ Most of the tumors in this study had invaded the upper trunk of the brachial plexus. However, separate research has shown that the lower trunk is the classical location for lipomatous compression of the brachial plexus.^1–3^

The symptoms of compression created by a lipoma within the brachial plexus could best be defined as either Neurologic Thoracic Outlet Syndrome (NTOS) or a “brachial plexopathy,” with the later imposing similar symptoms to NTOS, depending on the location of the mass.^2^ The pathophysiology of NTOS is due to neural compression, usually by a mass impinging on the brachial plexus or due to extracorporeal force causing fracture of the first rib resulting in neural compression.

Although arterial thoracic outlet syndrome (ATOS) and venous thoracic outlet syndrome (VTOS) are two separate entities, they are often classified together as “vascular” forms of thoracic outlet syndrome.^4^ NTOS typically presents as weakness and degeneration of the hand muscles at the base of the thumb and forearm finger flexor muscles, sometimes presenting with neck pain. In contrast, arterial thoracic outlet syndrome presents with symptoms of ischemia and venous thoracic outlet syndrome including limb swelling, discoloration, and pain.^4–6^ In more severe NTOS cases, upper extremity “shooting” pain or paralysis may occur.^2,6^

Due to their similar symptomatology, the clinical diagnosis of NTOS can be very difficult and often mistakenly classified as arterial thoracic outlet syndrome.^4^ Along with a comprehensive patient history, a thorough physical exam including neck rotation and head-tilting assessments are especially important to fully diagnose NTOS.^4^

Numerous surgical and non-surgical treatments for NTOS can be effective and helpful in both the short and long-term,^7^ although physical therapy has remained the first choice treament.^8^ Lifestyle modifications are also usually recommended, with reduction of overhead stress being paramount.^8^ Patients diagnosed with NTOS may also find relief from pain control strategies (e.g., anti-inflammatory medication, muscle relaxants, and botulinum injections).^8^ NTOS surgical treatment can be successful in many cases, with most patients experiencing a reduction in symptoms.^6,9,10^

## EDUCATIONAL CASE PRESENTATION

In 2018, the authors at the Michigan State University Prosection Lab identified the tumorous mass postero-inferior to the right clavicle in the body of a male cadaver donor in his 70s-80s. The lateral location and depth of this mass was considered atypical; and because it compressed a brachial plexus nerve, the authors strongly believed that the mass would have presented an alternative set of symptoms not typically described by individuals with NTOS. To gather more information, the authors retrospectively investigated the donor’s clinical history to determine what kinds of documented symptoms this man had experienced and/or whether he had received any conservative or surgical intervention.

### Initial Discovery: Tumorous Mass

**Figure 1:**
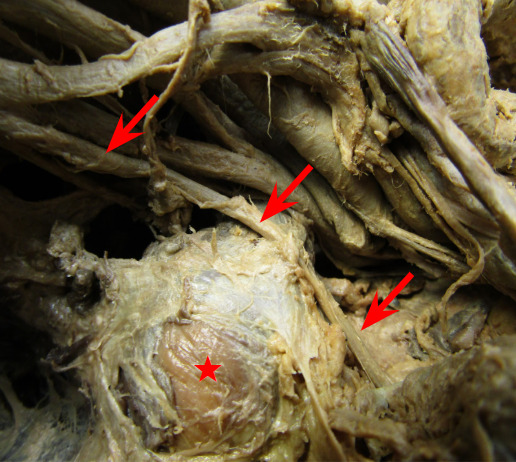
The suprascapular nerve (red arrows) was bent, most likely due to compression by the lipoma (red star).

**Figure 2:**
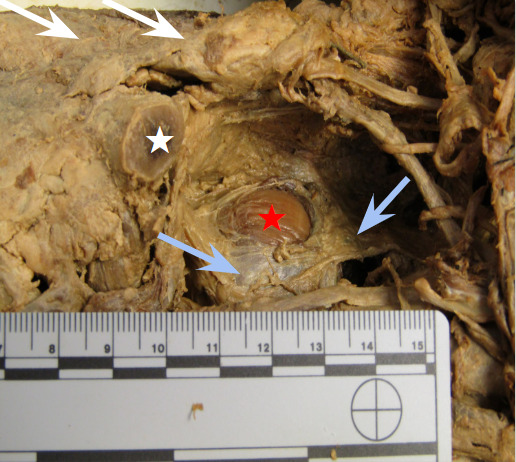
Location of the lipoma (red star) postero-inferior to the clavicle (cut, white star), and deep to the trapezius muscle (blue arrows). The mass was encapsulated in a membranous-like tissue.

During prosection of the male cadaver donor, a preclerkship medical student (i.e., first author RM) initially discovered a large rounded doughy off-white colored tumorous mass measuring 2.6 cm. x 2.2cm. The mass was located deep to the trapezius muscle and infero-posterior of the right clavicle. Further investigation by the team of authors showed that it was seated lateral to the suprascapular nerve and clearly exerted enough tension on the nerve to cause a visible sag. (Figure 1) Additionally, a membranous-like fascia surrounded the mass and extended out to attach anteriorly to the second rib and posteriorly to the superior angle of the scapula (Figure 2).

After consulting the Willed Body Program director to obtain permission for advanced investigations and data collection, the student (RM) proceeded with further investigation and processing of the mass under the mentorship of the pathologist (author PK), anatomy faculty (author LK) and prosection specialist (author WM).

**Figure 3:**
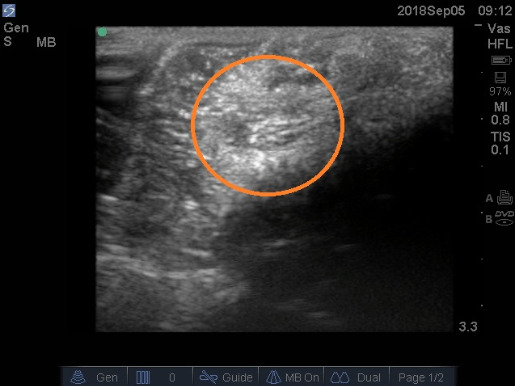
Ultrasound image of the lipoma (outlined by orange circle) before removal. Amplification was drastically increased to provide this image, and it is likely that this mass would not have appeared as clearly under typical ultrasound procedure in the living person.

The mass was photographed in situ (i.e., in it its original position) from various angles, and imaged using 13-6 MHz ultrasound transducer powered by Sonosite M-Turbo Ultrasound machine (FuJIFILMS Sonosite, Inc). The ultrasound images confirmed the round outline of the mass with no evidence of fluid inside (Figure 3).

**Figure 4:**
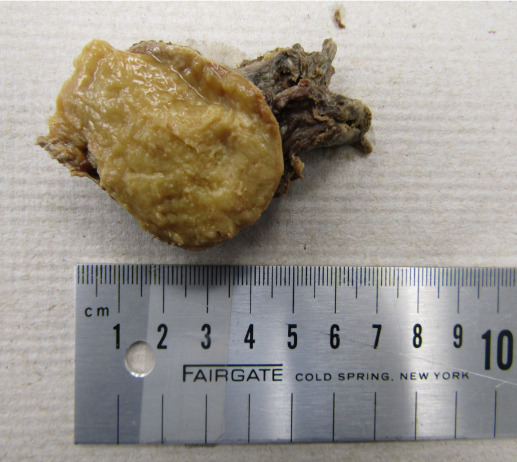
Cut surfaces of the lipoma are uniform and soft, with a compact and cheesy-like center. The mass expanded in size once half of it was removed from the donor.

**Figure 5:**
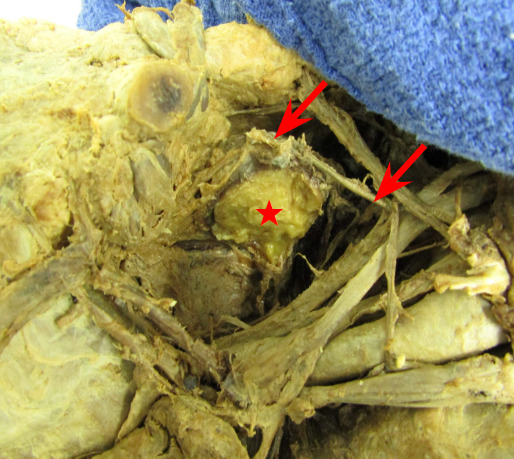
The relaxation of the suprascapular nerve (red arrows) was evident after half of the lipoma (red star) was sectioned off.

Based on advice of the pathologist, half of the mass was also sectioned off and sent to a histopathology lab for analysis. The other half of the mass remained in-situ and permitted the researchers to document that the interior of the mass was compact and cheesy-like in appearance (Figure 4). After half of the mass was removed, the tension on the suprascapular nerve was overtly relieved (Figure 5).

### Subsequent Follow-Up: Histopathological Investigation

**Figure 6:**
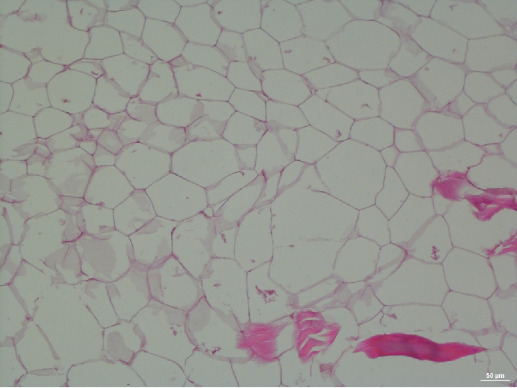
Histopathologic section of the mass (H&E stain) indicating lipoma. The adipocytes are noted to be the empty-looking cells in the center and left side of the image, as fatty tissues do not readily stain with H&E. Note that the nucleic acid content of the adipocytes was largely lost due to the embalming fluid used for cadaver preservation.

Upon suggestion of the pathologist author, the sections were stained by hemotoxylin and eosin (H&E) stain (Figure 6). It was evident that the mass consisted of only mature adipocytes (i.e., fat cells), with no pathological evidence of cellular abnormality, tissue necrosis, or increased mitotic activity (i. e, cell division). Therefore, the mass was classified as a benign lipoma. No apparent metastatic growths were found anywhere else in the donor cadaver.

In addition to data acquired from the Director of the Willed Body Program, the authors consulted with the donor’s family to obtain further information from his clinical history. Notably, this individual had indeed reported chronic pain and discomfort of his shoulder and upper extremity. The patient had not had a prior history of surgery or other conditions but had received some form of physical therapy. Despite his symptoms, this donor had lived a very active lifestyle and was involved as a coach and player in numerous physical sports, hunting and fishing. The fact that he played sports and worked as a coach probably placed him at further risk for the development of NTOS, as repetitive movements and physical strains have been associated with increased incidence of NTOS.^4,8^

## DISCUSSION

Given its size, position and the tensile stress this lipoma exerted on this man’s supraclavicular nerve, this patient likely experienced neuropathic symptoms consistent with NTOS involving this nerve and his lateral cord of the brachial plexus. Alternatively, this mass may have caused brachial plexopathy masquerading as symptoms of NTOS.

Although it was impossible for the authors to fully confirm specific symptoms pertaining to this lipoma, it was logical for them to concluded that this individual had likely experienced weakness of supraspinatus and infraspinatus (i.e., upper back muscles) and possibly deltoid and biceps with dysesthesias (i.e., itching, burning) extending into the upper arm and shoulder.

There is a high probability that the donor’s active lifestyle contributed to more physical activity and physical injury, which is one of the most common causes of the NTOS-like symptoms.^4^ It is important to note that the spatial location of the tumor may have led to misdiagnosis of the cause of brachial plexopathy, although the exact reason the patient never sought ongoing treatment for his symptoms remains unanswered.

Because NTOS symptomatology can be highly variable, diagnosis is often very difficult.^11^ In addition, evidence-based clinical criteria for a NTOS diagnosis have not been established, with diagnostic processes usually quite multifaceted.^12,13^ In this man’s case, the authors question whether the slow-growing nature of his atypically located mass might have made a definitive diagnosis more difficult.

As suggested in this case, routine ultrasound imaging may be largely ineffective in identifying thoracic lipomas causing NTOS. Additionally, electromyography (EMG) and nerve conduction velocity tests for these patients are often normal.^14^ X-ray films can be helpful when identifying a pathologic first rib (i.e., a rib which forms above the typical rib 1, causing compression of the brachial plexus), but are otherwise ineffective when diagnosing NTOS.^14^ Magnetic resonance imaging (MRI) may be a more helpful imaging modality used to visualize soft-tissue abnormalities such as thoracic outlet borders.^14^ A physical exam is often the most helpful procedure when attempting to evaluate possible NTOS.^14^

In certain types of NTOS, surgical treatment directed at scalene resection is performed to lessen brachial plexus irritation and alleviate pressure on the nerves. Although no standard surgical approach has been identified, various surgical approaches (i.e., transaxillary, supraclavicular, or subclavicular) can be considered based upon the location of the mass requiring removal.^3,15,16^ These surgical methods each have demonstrated similar degrees of success, with most postoperative patients discharged the following day with minimal side effects or complications.^8^

This case highlights the academic value of anatomy and prosection labs in medical school curricula already found in other settings.^17–19^ Although recent research has demonstrated that both approaches contribute to anatomical learning, individual and group dissections can offer unique educational benefits for anatomical and educational outcomes by providing medical students with improved anatomical knowledge, understanding of anatomical relationships, and clarification of difficult anatomical concepts.^17–22^

However, medical students have described a range of emotions (e.g., anxiety, fear, excitement) in anticipation of anatomy lab experiences.^23,24^ Upon completion of their anatomy lab experiences, students can have overwhelming positive experiences, including a sense of accomplishment.^23^ Cadaver donors can serve as a student’s “first patient,” teaching them respectful professional behaviors and offer them ways to cope with death and dying.^25^

During recent years, more medical school curricula have entirely moved away from the use of cadavers in favor of other learning methodologies.^26^ Unquestionably, full-body dissection is time intensive and students can often suffer from an especially steep learning-curve.^25^ Nevertheless, the use of short term directed exposures to anatomy dissection processes can offer motivated students clinically relevant self-directed and peer teaching experiences, particularly for those interested in surgical techniques and specialties.^22,25,27^ As in this case, exercises to scrutinize pathological entities also provide anatomy students with a tangible sense of the diagnostic skills that will be required of them as prospective healthcare professionals.^28^

## CONCLUSIONS

This medical education report is provided to demonstrate two key medical education examples: the clinical learning acquired after a prosection lab medical student identifies an unusually located mass and description of how human prosection and anatomy labs resources can be successfully utilized to enhance the education of medical students.

During the coming years, medical students who wish to augment their requisite coursework activities can ideally continue elective participation in anatomy and prosection laboratories. When ambitious medical students derive anatomic information and findings from such labs using investigative methodologies, their critical thinking insights can exceed those typically inherent in medical education settings.

### Funding

The authors report no external funding source for this study.

### Conflicts of Interest

The authors declare no conflict of interest.

## Acknowledgements

The researchers express extreme gratitude to the family of the Donor for consent, and to Jacque Liles, MSW, Director of the MSU Willed Body Program and Director of Anatomical Resources, Gross Anatomy Labs, without whom this project could not have been completed.

## Donor Consent

Living relatives of the donor gave informed consent to study the donor and to publish any findings and photographs without personal identifiers.

Submitted for publication July 2020.

Accepted for publication August 2020.
